# 

**Published:** 1908-12

**Authors:** 


### CONTENTS.

ORIGINAL COMMUNICATIONS—

Surgical Conscience. By R. M. Harbin, M. D., Rome, Ga. .... 461

Active Exercises in the Treatment of Locomotor Ataxia. By Theodore Toepel, M. D., Atlanta, Ga. .... 468

Malaria. By D. H. DuPree, B. S., M. D., Athens, Ga. .... 474

Tracheobronchoscopy and Oesophagoscopy. By Otis H. Johnson, B. A., M. D., Athens, Ga. .. 477

Etiology, Infection Carries, and Treatment of Typhoid Fever. By B. W. Hall, M. D., Bowman, Ga. .... 481

EDITORIALS .... 489

NEWS AND NOTES .... 498

SELECTIONS AND ABSTRACTS .... 500

BOOKS RECEIVED .... 503

BOOK REVIEWS .... 505

MEDICAL ITEMS .... 509

